# Diuretic Renography in a Sitting Position with F + 10(sp) Method for Diagnostic Management of Primary Megaureter in Adults

**DOI:** 10.1055/s-0045-1802665

**Published:** 2025-02-20

**Authors:** Girolamo Tartaglione, Francesco Pio Ieria, Riccardo Bertolo, Pierluigi Bove, Matteo Vittori

**Affiliations:** 1 Department of Nuclear Medicine, Cristo Re Hospital, Rome, Italy; 2 Department of Surgery, Dentistry, Pediatrics and Gynecology, Urology Unit, University of Verona, Verona, Italy; 3 Department of Urology, San Carlo di Nancy Hospital, Rome, Italy

**Keywords:** diagnostic imaging, furosemide, megaureter, radioisotope renography, sitting position

## Abstract

**Objective** Primary megaureter is a disease defined as the dilatation of the ureter caused by a congenital abnormality of the lower ureteral tract. Adult patients with primary megaureters typically present with no symptoms, making conservative management the preferred treatment. However, if an obstruction is present, we recommend ureteral reimplantation. The major diagnostic challenge is to distinguish which patients need surgical intervention. Ultrasound, computed tomography, and magnetic resonance imaging urogram findings of obstruction may be misleading because they are based on morphological aspects, and persistence of contrast in the upper urinary tract is not specific for obstruction. Renal scintigraphy is the key test for choosing surgical or conservative treatment; historically, the criterion for surgical treatment is the decrease of split renal function (SRF) less than 40%. Unfortunately, SRF might be only an indirect finding of obstruction; otherwise, the 20-minute/peak ratio may offer urologists an earlier, direct, and reliable index of urine outflow in monitoring ureteral flow. This study suggests that the F + 10(sitting position) test, which measures diuretic renography (DR) in a sitting position, is a new and useful way to find out how well primary megaureters are working for diagnosing and treating them. It focuses on the 20-minute/peak ratio that can be found when gravity is favorable.

**Methods** Twenty-eight adult patients (15 males, 13 females) affected by primary megaureter were retrospectively enrolled. Twenty-six patients had unilateral megaureter, and 2 patients had bilateral megaureter, for a total of 30 megaureters radiologically confirmed (16 left, 14 right). In total, we performed twenty-eight 99mTc-mercaptoacetyltriglycine DR in a sitting position using the F + 10 (sitting position) method. In our series, 17 patients received conservative treatment, and 11 patients underwent ureteral reimplantation.

**Results** Based on the 20-minute/peak ratio values, 17 out of 30 megaureters were diagnosed as obstructed. A discordance between SRF and 20-minute/peak ratio findings has been seen. No side effects were seen.

**Conclusion** A decrease in SRF is an indirect and late index of obstruction. Twenty-minute/peak ratio measured by DR in sitting position may improve the sensitivity and accuracy of the test for diagnosis of obstructive megaureters.

## Introduction

Primary megaureter is the second most common cause of hydronephrosis in newborns, the incidence of obstructed megaureter is 1 per 10,000 population. 1 A primary megaureter is a ureter dilation with a diameter greater than 7 mm. 2 This complication is due to a congenital developmental defect in the muscular layer of the ureter, which can be complete or segmental. Ultrasound may easily reveal ureteral dilation, but ultrasonography is only descriptive and supplies no details on renal function or drainage. We recommend long-term follow-up of conservatively managed primary megaureters due to evidence of late recurrence in teenage and adult patients. 3 The need for a correct assessment of ureteric function in patients with a dilated ureter has increased, given that approximately 20% of patients may present with urinary tract infection and abdominal pain. 4 Many patients with megaureter often present without any symptoms and have a normal serum creatinine level. 5 Voiding cystography may easily detect the presence/absence of vesicoureteral reflux in the megaureter. 6 7 If there is no obstruction, the recommended treatment is conservative management; if not, it is surgical treatment. For patients with primary megaureter, we recommend renography. Nuclear medicine labels any obstructed ureter with a delay in radiotracer drainage. However, when performing imaging in the supine position, urine stasis in the dilated ureter could potentially mislead the test, even in the absence of obstruction. Currently, a decrease in split renal function (SRF) less than 40% is considered the criterion for surgical treatment. 5 We calculate SRF by dividing the radioactive tracer accumulation from each side in the first 2 minutes of the renographic curve by the total accumulation in both kidneys in the same period. The relative function is expressed as a percentage of the overall function. A decrease in SRF% may indicate a loss of kidney function. 8 9 10 Regrettably, other authors have confirmed that even in the presence of partial or no drainage, the SRF may not be significantly impaired. 11 12 To find out how well primary megaureters are working, we performed diuretic renography (DR) with the F + 10(sitting position [sp]) method, while sitting to measure the direct urine outflow indices after giving a diuretic in a gravity-favorable environment. 13

## Materials and Methods

We obtained the results by retrospectively observing patients who visited our nuclear medicine center between 2016 and 2023. We studied 28 adult patients, 15 males and 13 females, with a median age of 42 years (range: 18–73) and diagnosed them with primary megaureters based on radiological findings such as dilatation of the ureter with a diameter greater than 7 mm and stasis of contrast in the ureter. Twenty-six patients had a unilateral megaureter, and two patients bilaterally (16 left megaureters, 14 right megaureters). Seventeen patients received conservative treatment, while 11 patients underwent surgical treatment. A significant obstruction or progressive loss of kidney function was indications for intervention. All patients who had routine micturating cystography or cystoscopy with visualization of the ureteral orifices did not have significant reflux. We performed 28 DR with 99mTc-mercaptoacetyltriglycine (MAG3) using the F + 10(sp) method, examining the patient in a sitting position. Due to clinical needs, a subgroup of five patients (4 males and 1 female) underwent DR several times for follow-up with adjunctive 15 exams. This study followed the national guidelines set by the Italian Association of Nuclear Medicine, AIMN (v. 03/2017) for the F + 10(sp) method. Written informed consent was waived. We stopped angiotensin-converting enzyme inhibitors and diuretics 48 hours before the test. We asked all patients to empty their bladders before the scan. We studied patients under normal hydration conditions. Before the test, we measured blood pressure and weight to calculate the right dose of furosemide (0.25 mg/kg) for intravenous delivery. We used a dual-detector, large-field-of-view gamma camera (General Electric, Infinia Xeleris III system) with a free geometry gantry design, enabling 0-, 90-, and 180-degree orientations of the detectors, allowing scanning at all positions. We positioned a detector vertically, tilting it 90 degrees toward the posterior view. The camera reached a peak at 140 keV with a 20% window. We used a low-energy general-purpose collimator, as the purpose of the study was to increase the sensitivity to better quantify radioactivity in the kidneys, ureters, and bladder. We seated the patient in a suitable imaging chair providing sufficient support to prevent movement during the study. During dynamic acquisition, we aligned the shoulders and pelvis with the patient's back facing the detector. To improve resolution and reduce scatter, we minimized the distance between the detector and the patient's back. The field of view included the kidneys, heart, and bladder. After putting in an intravenous catheter with an injection valve, we gave a bolus dose of 150 to 200 MBq of 99mTc-MAG3 diluted in 0.2 to 0.3 mL at time 0'. This was followed right away by flushing with 1 to 2 mL of normal saline solution. We acquired images from the posterior view starting when the radioactive bolus was in the mediastinum. According to the protocol, we obtained a 20-minute dynamic scan with a frame rate of 2 sec/frame for the first 60 frames and 10 sec/frame for 108 frames, using a 128 × 128 matrix and zoom ×1. At the fifth minute, the patient drank 400 to 500 mL of water. During dynamic acquisition, we administered diuretics intravenously 10 minutes after tracer injection. We injected a furosemide dose of 0.25 mg/kg (range 10–20 mg). However, we systematically adjusted the furosemide dose to 10 mg in patients whose systolic blood pressure was lower than 105 mm Hg. We checked the blood pressure in patients with persistent hypotension throughout the test. We applied an ice pack to the hand only if it was strictly necessary, as cold conditions tend to release catecholamines into the body, thereby preventing orthostatic hypotension. We acquired postvoiding images in the seated and supine positions at 20 and 60 minutes after the tracer injection, respectively, to complete the test. Two independent observers separately processed the data. A cine of raw data dynamic images with two locators was displayed for assessing patient motion. Following the automatic dynamic motion correction processing, the user drew the master kidney regions of interest (ROIs), as well as the ROIs for the aorta, bladder, and collecting system. We semiautomatically generated the kidney, background, and cortex ROIs using a commercial software (Xeleris 3.1 release, GE). We measured several indices: SRF%, also known as differential renal function, determines the relative contribution of each kidney to total renal function by comparing the first to second minute tracer accumulation. The normal range for renal activity is 45 to 55. 11 *T* max is the time it takes for the renogram to reach its highest level of activity (normal value: < 6 minutes). Diuretic half-time ( *T* 1/2) is the time between giving furosemide and the half-time of renal time-activity curve measured in sitting position (normal value: < 8 minutes); and the 20-minute/peak ratio is the ratio between the average activity of the curves from minutes 19 to 20 and the peak activity (normal value: < 0.25). The 20-minute/peak ratio value, measured in conditions of favorable gravity, may allow a clear and objective distinction between obstructed and normal kidneys, as verified in our previously published works. 13 14 15 Accordingly, we considered the 20-minute/peak ratio value to be the gold standard for obstruction diagnosis.

## Results

We evaluated 28 DRs. Thirty megaureters (16 left, 14 right) were considered suspected obstructive megaureters, based on radiological findings. Table 1 displays demographic, clinical data, and main functional indices for surgically treated patients, while Table 2 displays the same information for conservatively treated patients. We diagnosed 17 out of 30 megaureters as obstructed (56.6%) based on the values of the 20-minute/peak ratio. We found a discrepancy between the SRF% and the 20-minute/peak ratio findings ( Table 3 ). Table 4 expresses the sensitivity, specificity, disease incidence, positive and negative predictive values, and accuracy of the main renal functional indices (SRF%, *T* max, and diuretic T1/2) in percentage terms. Adjunctive 15 DRs were performed as follow-up in a subgroup of 5 patients ( Table 5 ). Two independent observers processed all the data. The agreement was 97.6%, and Cohen's *k* was 0.94. During the test, we measured an average blood pressure drop of 10 mm Hg. We observed no side effects such as orthostatic diuretic-related hypotension, bladder fullness, or disruption of the test due to voiding.

**Table 1:** Demographic, clinical data, and comparison between SRF and 20-minute/peak ratio in patients surgically treated

Patient #	Sex	Years	Creatinine (mg/dL)	L SRF (%)	L 20-minute/peak ratio	R SRF (%)	R 20-minute/peak ratio	Diagnosis
1	F	62	1.20	66	0.26	34	0.37	Right megaureter
2	M	20	1.13	40	0.80	60	0.20	Left megaureter
3	F	65	0.76	61	0.20	39	0.53	Right megaureter
4	F	41	0.80	48	0.19	52	0.22	Left megaureter
5	M	41	1.20	64	0.12	36	0.89	Right megaureter
7	F	18	0.82	52	0.17	48	0.24	Right megaureter
15	M	43	1.10	48	0.21	52	0.18	Right megaureter
17	F	54	0.93	36	0.27	64	0.15	Left megaureter
20	M	50	1.00	45	0.18	55	0.15	Left megaureter
21	M	51	1.84	46	0.09	54	0.26	Right megaureter
24	M	23	1.19	28	0.06	72	0.09	Left megaureter

Abbreviations: F, female; L, left; M, male; R, right; SRF, split renal function.

**Table 2:** Demographic, clinical data, and comparison between SRF and 20-minute/peak ratio in patients conservatively treated

Patient #	Sex	Years	Creatinine (mg/dL)	L SRF (%)	L 20-minute/peak ratio	R SRF (%)	R 20-minute/peak ratio	Diagnosis
6	M	53	0.85	45	0.18	55	0.21	Left megaureter
8	F	39	1.20	54	0.21	46	0,19	Right megaureter
9	M	42	0.81	66	0.10	34	0.24	Right megaureter
10	M	29	1.69	40	0.62	60	0.30	Bilateral megaureter
11	M	52	0.69	76	0.21	24	0.74	Right megaureter
12	F	51	0.75	52	0.45	48	0.22	Left megaureter
13	F	47	0.90	53	0.21	47	0.20	Left megaureter
14	M	21	0.96	44	0.15	56	0.21	Bilateral megaureter
16	F	65	1.20	20	0.38	80	0.21	Left megaureter
18	M	42	1.20	26	0.47	74	0.26	Left megaureter
19	F	43	0.59	56	0.26	44	0.20	Left megaureter
22	F	57	0.84	38	0.53	62	0.23	Left megaureter
23	M	23	1.06	44	0.19	56	0.14	Left megaureter
25	M	31	1.19	66	0.17	34	0.45	Right megaureter
26	M	45	1.00	49	0.50	51	0.23	Left megaureter
27	F	45	0.89	49	0.17	51	0.27	Right megaureter
28	F	38	0.90	50	0.17	50	0.14	Left megaureter

Abbreviations: F, female; L, left; M, male; R, right; SRF, split renal function.

**Table 3:** Univariate analysis of variables (sex, side, SRF%, 20-minute/peak ratio) that predict obstruction in 30 renal unit (RU) with radiologically diagnosed obstructive megaureter

Variable	RU No obstruction ( *n* = 13)	RU obstruction ( *n* = 17)	*p* -Value
Sex (male/female)	8/8	8/6	NS
Side (left/right)	8/5	9/8	NS
SRF% (Nv = 45–55)	45.62 (SD 7.56)	40.53 (SD 11.60)	NS
20-minute/peak ratio (Nv < 0.25)	0.18 (SD 0.04)	0.48 (SD 19.43)	< 0.0001

Abbreviations: NS, not significant; SD, standard deviation; SRF, split renal function.

**Table 4:** A comparison of statistic values of main renal functional indices SRF%, *T* max, and diuretic T1/2, considering 20-minute/peak ratio > 0.25 as suggestive for diagnosis of obstruction

Statistic	SRF%	*T* max	Diuretic T1/2
Sensitivity	64.71%	88.24%	75.00%
Specificity	76.92%	30.77%	85.71%
Positive likelihood ratio	2.80	1.27	5.25
Negative likelihood ratio	0.46	0.38	0.29
Disease prevalence	56.60%		
Positive predictive value	78.53%	62.44%	87.26%
Negative predictive value	62.56%	66.73%	72.44%
Accuracy	70.01%	63.30%	79.65%

Abbreviation: SRF, split renal function.

**Table 5:** In the subgroup of patients who had multiple diuretic renography procedures as part of a follow-up, demographic, clinical, and primary functional indices data were collected

Patient #	Sex	Years	Conservative/Surgical (C/S)	Creatinine (mg/dL)	L SRF (%)	L 20-minute/peak ratio	R SRF (%)	R 20-minute/peak ratio	Diagnosis
2	M	20	S	1.13	40	0.80	60	0.20	*Left megaureter* * *stent jj*
2a		21		1.40	29	0.85	71	0.08	
2b		22		1.10	25	0.87	75	0.26	
2c *		23		1.10	16	0.58	84	0.15	
2d *		23		1.11	25	0.79	75	0.23	
3	F	65	S	0.76	61	0.20	39	0.53	*Right megaureter*
3a		66		0.85	67	0.22	33	0.60	
3b		67		1.39	62	0.30	38	0.70	
3c		73		1.09	58	0.31	42	0.89	
9	M	42	C	0.81	66	0.10	34	0.24	*Right megaureter*
9a		42		0.81	69	0.09	31	0.16	
9b		44		0.82	69	0.08	31	0.11	
21	M	51	S	1.84	46	0.09	54	0.26	*Right megaureter*
21a		55		1.30	44	0.28	56	0.51	
24	M	23	S	1.19	28	0.06	72	0.09	*Left megaureter*
24a		25			30	0.09	70	0.09	
24b		26			27	0.08	73	0.09	
24c		28			28	0.12	72	0.13	
24d		32			27	0.10	73	0.13	
24e		34			28	0.10	72	0.09	

Abbreviations: F, female; L, left; M, male; R, right; SRF, split renal function.

Note: Data from the first examination and data from later exams were compared.

* indicates the presence of a jj ureteral stent.

## Discussion

Today, a more conservative approach to treating primary megaureters is becoming more common. The purpose of urological imaging is to quickly separate the kidneys that do not need surgery from those that do. Approximately 30% of patients with primary megaureters will require intervention due to either functional imaging-based diagnosis of obstructive uropathy progression or clinical indications, including breakthrough urinary tract infection. 16 Computed tomography urography (CTU) and magnetic resonance imaging are equivalent to renography for measuring SRF%, but urine drainage and transit time are difficult to evaluate with radiological methods.

Some authors associated CTU protocols with a split-bolus dual-phase protocol, which includes furosemide (10–15 mg). This approach obtained the best dilation of the collecting system and distal ureter, reducing examination time and radiation exposure. However, the large volume of contrast medium administered at a flow rate of 2.5 mL/s, combined with the supine position, resulted in a continuous presence of contrast in the megaureter, making the CTU unable to distinguish between obstruction and dilation. This could potentially result in incorrect diagnoses even when there is no obstruction present. 17 18

Instead of CTU, renography has the advantage of injecting a minimum volume of radiotracer (0.2–0.3 mL) as a bolus. Normally, the radioactive bolus passes from the bloodstream through the kidneys and ureters to the bladder in around 20 minutes; this is the reason for which, in many centers, data are collected for 20 minutes after a tracer injection. 19

To date, renography is considered the best choice for the management of primary megaureters due to the lack of a suitable alternative for urine drainage assessment. The kidneys rapidly clear the tracer from the blood and excrete it via active tubular secretion and glomerular filtration following the intravenous injection of 99mTc-MAG3. We generated the 20-minute kidney and aortic time-activity curves from their respective ROIs. In clinical practice, the diagnosis is based on the SRF% level as well as a visual assessment of a late postvoiding scan. Some authors suggested injecting furosemide, but due to the supine position and various methods used, the diagnosis of obstructive megaureter remained subjective. 20 21 22 23 24 25 26 27 The British Association of Paediatric Urologists consensus meeting decided that since there is not a clear line between partial and good drainage, surgery should only be considered if the SRF percentage is less than 40% and there is massive or progressive hydronephrosis or a decrease in differential function on serial renograms. 5 A reduction of SRF%, calculated during the parenchymal phase of renography, may indicate a loss in kidney function. Other writers, however, have attested to the possibility that SRF can be normal even when there is aberrant drainage, and vice versa, that because comparing the radioactive tracer accumulation from each side in the first 2 minutes SRF is a measure of tracer input rather than output. 11 12 To date, despite significant advancements in imaging, there is still no reliable assessment of drainage, as the megaureter is a large capacity conduit that retains urine. In a recent systematic review on conservative management of primary megaureters based on changes in SRF%, Buder et al 28 suggested that future studies should overcome imaging methods' limitations by using standardized, comparable criteria and precise reporting of quantitative outcome data.

In our study, we suggest overcoming this diagnostic limitation by evaluating patients by DR in sitting position with the F + 10(sp) method.

The patient typically undergoes DR in a supine position, and the Society of Nuclear Medicine and Molecular Imaging-European Association of Nuclear Medicine recommends an adult dose of intravenous furosemide of 0.5 mg/kg, while the American College of Radiology–Society of Pediatric Radiology recommends 0.5 to 1.0 mg/kg, with variable timing and agreement on a maximum dose of 40 mg in healthy adults ( Table 6 ). 29 30

**Table 6:** A comparison of the many diuretic renography techniques

Method	Hydration	Position	Furosemide dose	Timing
F + 20	500 mL oral water at 20 min	Sitting or supine a	0.50 mg/kg (max 40 mg)	+20 min
F-15	500 mL oral water	Sitting or supine a	0.50 mg/kg (max 40 mg)	–15 min
Well-tempered	15 mL/kg/min saline solution IV in 30' + bladder catheter	Prone or supine a	1 mg/kg (up to 80 mg)	+20 min
F0	Oral water	Supine a	1 mg/kg (max 40 mg)	0 min
F + 10(sp)	500 mL oral water at 5 min	Sitting	0.25 mg/kg (10–20 mg)	+10 min

Abbreviations: IV, intravenous; sp, sitting position.

Note: The timing is the number of minutes that pass between injecting the tracer and administering the diuretic. The diuretic is administered a few minutes after the tracer injection, as indicated by F + , or a few minutes prior to the tracer administration, as indicated by F-.

a A later scan following voiding is recommended for a qualitative assessment of urine drainage because the urine output indices cannot be accurately quantified when the test is conducted in the supine or prone position.

It would be better to do DR while sitting down because this would stop the body from holding on to the radioactive urine. This could make it possible to get a good reading of the direct urine outflow indices, like the 20-minute/peak ratio or the diuretic T1/2.

However, it is crucial to emphasize that, when examining a patient in a sitting position, one should adjust the furosemide dose to prevent diuretic-related hypotension, considering the patient's body weight and systolic blood pressure. We administered a 0.25 mg/kg dose of furosemide (range: 10–20 mg). In patients with persistent hypotension and a systolic blood pressure lower than 105 mm Hg, we suggest injecting systematically a low dose of furosemide (10 mg). In our study, an adapted dose of furosemide with a suitable oral hydration volume of 400 to 500 mL demonstrated a high diagnostic value, avoiding the risk of diuretic-induced hypotension and bladder fullness-related problems without the need for a bladder catheter. We assessed 28 patients, of whom 11 underwent surgical treatment and 17 underwent conservative management.

Two tertiary referral hospitals performed the ureteroneocystostomy surgical treatment, either with or without ureteral tapering. Four patients underwent surgery with the full-thickness technique, and seven underwent surgery with the Lich–Gregoir technique. We performed all the procedures laparoscopically with robot assistance (Da Vinci Xi, Intuitive Surgical, Sunnyvale, California, United States). We did not observe any statistically significant differences in estimated blood loss, operative time, complications, or length of hospital stay between the two techniques.

**Fig. 1:**
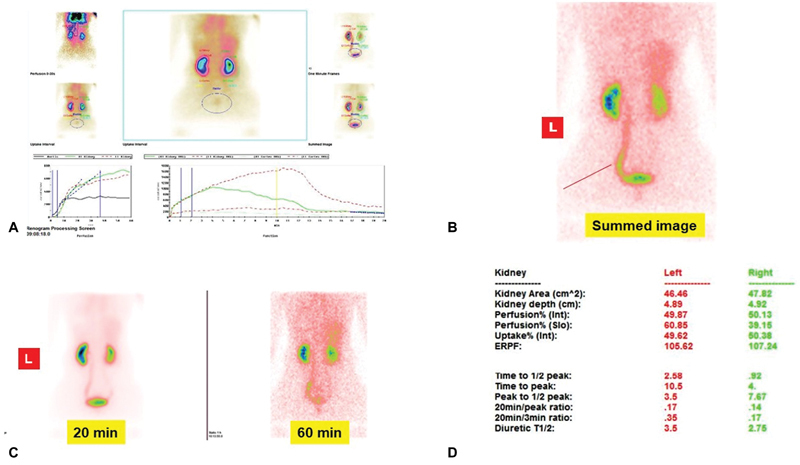
Female, 38 years, serum creatinine 0.90 mg/dL, left megaureter in conservative management with normal split renal function (SRF) % and outflow, ( **A** ) renogram, ( **B** ) summed image, ( **C** ) late scans, and ( **D** ) functional indices for side.

**Fig. 2:**
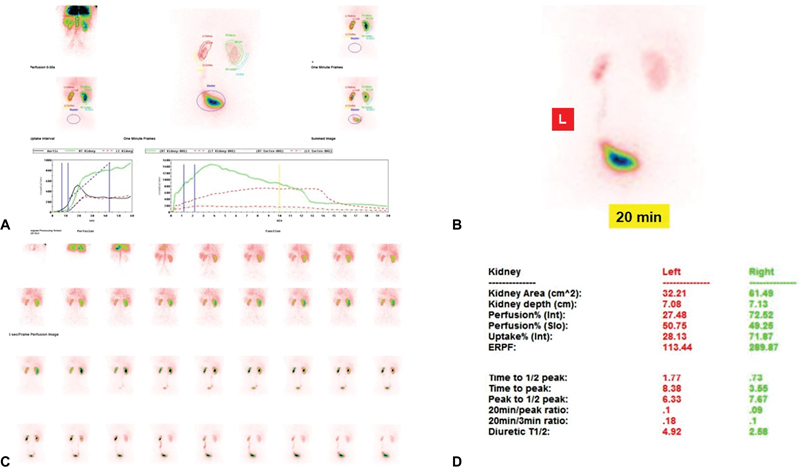
Male, 34 years, serum creatinine 1.1 mg/dL ureteroneocystostomy (UNC) left megaureter in follow-up with reduced split renal function (SRF) % and normal outflow, ( **A** ) renogram, ( **B** ) late scan, ( **C** ) dynamic images, and ( **D** ) functional indices for side.

**Fig. 3:**
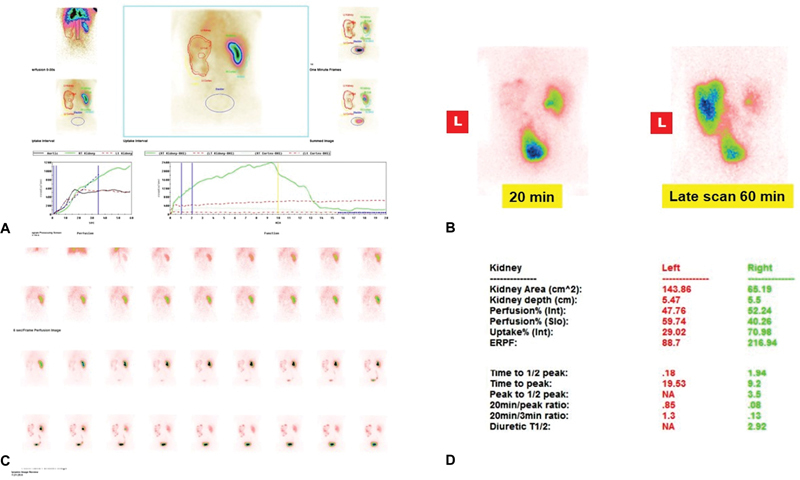
Male, 24 years, serum creatinine 1.2 mg/dL, ureteroneocystostomy (UNC) left megaureter with restenosis, ( **A** ) renogram, ( **B** ) late scans, ( **C** ) dynamic images, and ( **D** ) functional indices for side.

When examining patients in a sitting position, the 20-minute/peak ratio (normal value < 0.25) may allow us to clearly distinguish between nonobstructed and obstructed megaureters. By comparing the SRF% and the 20-minute/peak ratio, we found that the SRF% had a lower sensitivity for evaluating patients in the conservative management setting. A decrease in SRF% might be an index of decreased kidney function, and it may occur late after obstruction ( Fig. 1 ). In addition, the SRS% may remain unchanged in patients treated surgically despite a normalized outflow ( Fig. 2 ) or restenosis ( Fig. 3 ), as confirmed by follow-up data. Our experience suggests that the 20-minute/peak ratio may be more accurate than the SRF% for diagnosing obstructive megaureter in the early clinical stage, before renal function is impaired.

So, now that there are new minimally invasive techniques like robotic-assisted ureteroneocystostomy or other endoscopic ways to treat obstructive megaureter with low operative morbidity, we need a new tool to better measure transit time in the urinary tract. This creates the need for more accurate urological diagnostic imaging. 31 32 DR using the F + 10(sp) method while sitting down might give a more accurate evaluation of radioactive urine outflow by measuring the direct indicators of urine outflow when gravity is favorable. This is especially specific for diagnosing renal obstruction as soon as it develops.

The main limitation of the study is related to its single-center, retrospective design.

## Conclusion

To find out if someone has obstructive primary megaureters, the F + 10(sp) method with an adapted dose of furosemide is a noninvasive, safe, and well-tolerated test that can be used. We study patients without catheters in a more comfortable and natural setting.

A drop in SRF% is an indirect and late sign of obstruction; it does not seem to be very useful for diagnosing obstructive megaureter early on or checking on obstructive megaureters after surgery.

The 20-minute/peak ratio measured with the F + 10(sp) method may offer urologists an earlier, direct, and reliable index of urine outflow in monitoring ureteral flow, allowing prompt surgical intervention to avoid no restorable deterioration of SRF%.

That may improve the accuracy of diagnostic management of primary megaureter in adult patients, preventing renal impairment.
